# Calcification of breast artery as detected by mammography: association with coronary and aortic calcification

**DOI:** 10.3906/sag-1807-275

**Published:** 2019-02-11

**Authors:** Ayşegül İdil SOYLU, Korhan SOYLU, Ramazan AYDIN, Fatih UZUNKAYA, Kerim ASLAN, Ahmet Veysel POLAT

**Affiliations:** 1 Department of Radiology, Faculty of Medicine, Ondokuz Mayıs University, Samsun Turkey; 2 Department of Cardiology, Faculty of Medicine, Ondokuz Mayıs University, Samsun Turkey; 3 Clinic of Radiology, Kırıkkale Yüksek İhtisas Hospital, Kırıkkale Turkey

**Keywords:** Breast, coronary, aortic, calcification

## Abstract

**Background/aim:**

Coronary artery calcification (CAC) and aortic calcification (AC) are significant risk factors for coronary atherosclerosis. This study investigated how breast arterial calcification (BAC) detected from routine mammography correlates with coronary artery calcification and aortic calcification.

**Materials and methods:**

A total of 404 female patients above 40 years of age who, within a 6-month period, had undergone thoracic computed tomography and mammography for various reasons were screened retrospectively at our clinic. Mammographies were assessed for BAC and thoracic CT investigations were assessed for CAC and AC. Patients included in the study were scored as 0 (none), 1 (mild), 2 (moderate), or 3 (severe) depending on the number and shape of CAC, AC, and BAC lesions observed.

**Results:**

Four hundred and four females were enrolled in the study. While BAC was detected in 123 patients, no BAC was observed in the other 281 patients. In the BAC-positive patients, the rates of CAC (45.5% vs. 19.9%, P < 0.001) and AC (67.5% vs. 32.4%, P < 0.001) were notably higher than in the BAC-negative patients. In addition, multivariate regression analysis detected the presence of BAC as an independent variable for both CAC and AC.

**Conclusion:**

The presence of BAC appeared to be a significant risk factor for CAC and AC, and the BAC grade was considered an independent risk factor for CAC.

## 1. Introduction

Mammography is a screening tool used for investigating breast cancer in women above 40 years of age. The use of mammography for screening has been proven to provide significant advantages and it has been recommended by current practice guidelines. Breast artery calcification (BAC) is an incidental finding detected during mammography that is not associated with malignancy (1). Studies show that BAC is related to age (2), chronic renal disease (3), metabolic syndrome (4), and stroke (5). In addition, some studies support the notion that coronary atherosclerosis may be associated with BAC (6–8).

Coronary artery calcification (CAC) develops during the atherosclerotic process and affects plaque build-up. The calcification that occurs in the fibrous cap of an atheroma may facilitate the development of plaque rupture and a cardiovascular event (9). In addition, there is a well-established correlation between the presence of CAC and unfavorable events (10,11). In this study, the relation between BAC and atherosclerotic calcification (both coronary and aortic) was investigated and its correlation with other risk factors was assessed.

## 2. Materials and methods

### 2.1. Patient selection

Women above 40 years of age who had been assessed with both mammography and thoracic computed tomography (CT) within a 6-month period falling between January 2010 and December 2011 were assessed retrospectively. Patients who had received unilateral mastectomy were excluded due to the potential for missing possible calcification in the breast. A total of 404 women were included in the study. 

**Figure 1:**
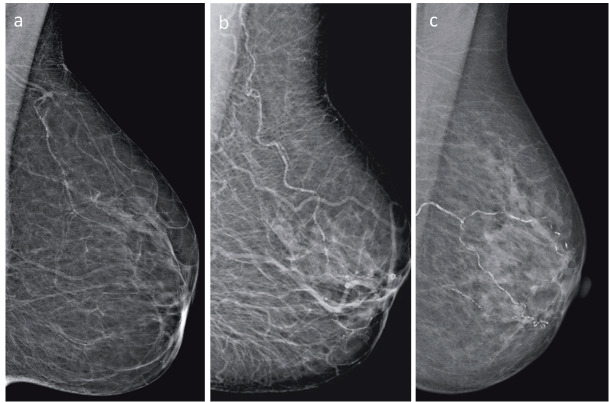
Breast artery calcification (BAC) score in mammography. Grade I small, punctate vascular calcifications (a); grade II coarse or tram-track calcifications, <3 vessels (b); grade III coarse or tram-track calcifications, ≥3 vessels (c).

Mammograms were obtained in a bilateral fashion using the Mammomat novation DR (Siemens, München, Germany) conventional mammography device at standard positions. Mammograms were evaluated for BAC at a work station by a radiologist with 5 years of experience in breast radiology. BAC was defined as vascular calcification in one or both breasts. The grade of BAC was determined by using a classification scale defined by Loberant et al. (12). Accordingly, absence of calcification was classified as grade 0; minor punctate vascular calcification as grade I; coarse, tram-track, or ring-type calcification in <3 vessels as grade II; and coarse, vascular calcification in ≥3 vessels as grade III (Figure 1).

All thoracic CTs obtained at appropriate time intervals for various etiologic reasons were included in the study. A thoracic CT 16-channel multislice CT device was used, with or without contrast, depending on the etiology. Imaging parameters included the following: 150 mAs, 120 kV, 2 × 16 mm collimation, pitch 1, reconstruction interval 1 mm, and tube rotation period 0.5 s. In order to evaluate the heart and the entire thoracic aorta, the field of view (FOV) was determined to include the space between thoracic entry and the 12th vertebra inferior. Slide thickness was determined on the basis of the etiologic cause and the results were obtained from a 3-mm section for patients with a preliminary diagnosis of pulmonary embolus and a 5-mm section for other thoracic CTs. CTs were evaluated for CAC and AC, and calcifications were scored. Vascular calcification was scored using a simple, predefined visual grading (13). Thoracic aorta calcification was measured at three different localizations as the ascending aorta (0–3), the descending aorta (0–3), and the supraaortic arteries originating from the arcus aorta (0–2). The measurements were then summed and scored as aortic calcification (0–8). On the calcification scale, the absence of calcification in the ascending or descending aorta was defined as grade 0, ≤3 foci as grade I, single calcification extending along 4–5 foci or ≥ 3 sections as grade II, and single calcification extending along >5 foci or ≥3 sections as grade III. For supraaortic arterial calcifications, absence of calcification was classified as grade 0, single artery calcification as grade I, and >1 arterial calcification as grade II. Coronary artery measurements were performed from the main coronary artery, left anterior descending artery, and right coronary artery; the values were then summed and a single number was obtained (0–12). The calcification scale defined absence of calcification as grade 0, presence of 1–2 calcification foci as grade I, single calcification extending >2 foci or ≥2 sections as grade II, and calcification along a long coronary artery segment as calcification grade III.

The protocol was approved by the institutional review boards and adhered to the Declaration of Helsinki. No informed consent was required for this retrospective study.

### 2.1. Statistical analysis

Statistical analyses were conducted using SPSS 15.0 for Windows (SPSS Inc., Chicago, IL, USA). Descriptive statistics were provided as mean, standard deviation, frequency, and percentage. The Kolmogorov–Smirnov test was used to evaluate whether continuous variables were normally distributed. Logarithmic transformation was performed for all nonnormally distributed variables. Student’s t-test was used to compare the two groups of values. A comparison of categorical values was carried out by means of the chi-square test. Any correlation between the data was tested with Spearman’s correlation analysis. Multivariate logistic-regression analysis was also performed, and the model included potential confounders (breast artery calcification, diabetes mellitus, age, hypertension, HDL-C, Hs-CRP, creatinine). While continuous data were expressed as mean ± SD (standard deviation), categorical data were expressed as percentage values, and P < 0.05 was accepted as statistically significant.

## 3. Results

### 3.1. Baseline clinical characteristics

A total of 404 women were included in the study. Breast artery calcification was detected in 123 patients, while 281 had no BAC. The baseline characteristics of both groups are presented in Table 1. While the groups were similar with respect to incidence of hypertension and diabetes, the BAC-positive patients were older (67.9 ± 9.9 vs. 54.5 ± 9.1 years, P < 0.001). In addition, Hs-CRP levels, HDL-C levels, and hemoglobin levels were all significantly different in the two groups. In 104 of the 123 patients with positive BAC (84.6%), BAC was bilateral; 10 had BAC (8.1%) in the right breast artery only and 9 had BAC (7.3%) in the left breast artery only. The calcification grade was grade I in 44 patients (35.8%), grade II in 40 patients (32.5%), and grade III in 39 patients (31.7%).

**Table 1:** Baseline characteristics.

	BAC-negative (n = 281)	BAC-positive (n = 123)	P
Clinical data			
Age, years	54.5 ± 9.1	67.9 ± 9.9	<0.001
Hypertension (%) (n = 243)	118 (42.0)	43 (35.0)	0.111
Diabetes mellitus (%)	50 (17.8)	25 (20.3)	0.319
Biochemical and hematological data			
Total cholesterol, mg/dL	197.7 ± 51.8	189.3 ± 59.1	0.209
Low-density lipoprotein, mg/dL	121.8 ± 45.6	116.7 ± 50.3	0.388
High-density lipoprotein, mg/dL	48.9 ± 20.1	42.7 ± 18.6	0.012
Triglyceride, mg/dL	143.5 ± 67.8	146.4 ± 78.2	0.751
Creatinine, mg/dL	0.78 ± 0.60	0.82 ± 0.35	0.410
Fasting glucose, mg/dL	114.3 ± 46.9	121.4 ± 57.6	0.236
High sensitivity CRP (mg/dL) (n = 286)	27.2 ± 42.3	45.5 ± 63.9	0.004
Hemoglobin, g/dL	12.1 ± 1.7	11.4 ± 1.9	<0.001
Breast artery calcification			
Right BAC		10 (8.1)	
Left BAC		9 (7.3)	
Bilateral		104 (84.6)	
Degree of breast artery calcification			
1		44 (35.8)	
2		40 (32.5)	
3		39 (31.7)	

BAC: Breast artery calcification, CRP: C-reactive protein.

### 3.2. Coronary calcification

**Figure 2:**
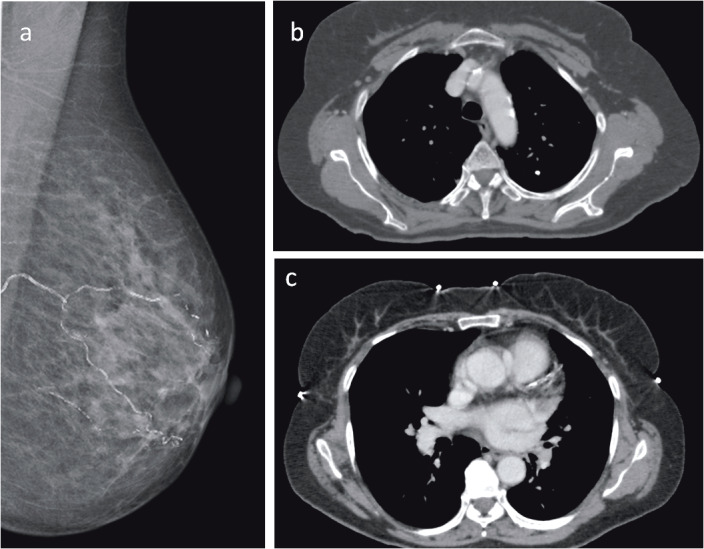
Mammography of a 62-year-old woman with grade III vascular calcification in the left breast: (a) while thoracic CT shows calcified plaques in arcus aorta (b) and left main coronary artery (c).

CAC was detected in 112 (27.7%) of the 404 patients included in our study. Evaluation for coronary calcification showed a notable difference between the two groups. CAC was detected in 56 of the BAC-negative patients (19.9%), while 56 of the BAC-positive patients (45.5%) also had CAC (P < 0.001) (Figure 2). This difference preserved its significance at all coronary localizations. With respect to CAC grade, there were more severe calcifications (grades II and III) in the BAC-positive group. Grade I CAC was present in a greater number of BAC-positive patients, but there was no statistical significance (Table 2).

**Table 2:** Vascular calcification of patients.

	BAC-negative (n = 281)	BAC-positive (n = 123)	P
Coronary calcification	56 (19.9)	56 (45.5)	<0.001
Localization			
LMCA	19 (6.8)	28 (22.8)	<0.001
LAD	48 (17.1)	49 (39.8)	<0.001
LCX	16 (5.7)	30 (24.4)	<0.001
RCA	15 (5.3)	30 (24.4)	<0.001
Degree of coronary calcification			
1	42 (14.9)	27 (22.0)	0.085
2	10 (3.6)	19 (15.4)	<0.001
3	4 (1.4)	10 (8.1)	0.001
Aortic calcification	91 (32.4)	83 (67.5)	<0.001
Degree of aortic calcification			
1	70 (24.9)	55 (44.7)	<0.001
2	19 (6.8)	19 (15.4)	0.006
3	2 (0.7)	9 (7.3)	<0.001
Localization of aortic calcification			
Supraaortic calcification	41 (14.6)	45 (36.6)	<0.001
Ascendant aortic calcification	27 (9.6)	46 (37.4)	<0.001
Descendent aortic calcification	71 (25.3)	68 (55.3)	<0.001

LMCA: Left main coronary artery, LAD: left anterior descending artery, LCx: left circumflex artery, RCA: right coronary artery.

### 3.3. Aortic calcification

In terms of AC, 91 BAC-negative patients were detected to have AC (32.4%) while 83 BAC-positive patients (67.5%) had AC (P < 0.001). With respect to the grade of AC, BAC-positive patients had more aortic calcifications at all grades.

### 3.4. Correlation and regression analysis

Assessment of the parameters that showed correlation with the CAC grade revealed that BAC grade, age, aortic calcification grade, triglyceride level, creatinine level, fasting glucose, and Hs-CRP level positively correlated with the CAC grade. On the other hand, HDL levels showed a negative correlation (Table 3). In addition, a weak correlation was detected between Hs-CRP and BAC (r = 0.205, P < 0.001).

**Table 3:** Correlation between coronary artery calcification and others parameters.

	Degree of coronary artery Calcification	
	R value	P
Degree of breast artery calcification	0.376	<0.001
Age, years	0.424	<0.001
Degree of aortic calcification	0.595	<0.001
Diabetes mellitus	0.162	0.001
Hypertension	0.089	0.072
Total cholesterol, mg/dL	–0.115	0.062
Low-density lipoprotein, mg/dL	–0.099	0.079
High-density lipoprotein, mg/dL	–0.180	0.001
Triglyceride, mg/dL	0.129	0.023
Creatinine, mg/dL	0.202	<0.001
Fasting glucose, mg/dL	0.170	0.001
High sensitivity C-reactive protein, mg/dL	0.143	0.016
Hemoglobin	–0.099	0.057

Multivariate regression analysis showed that BAC (OR 1.647; 95% CI 1.103–2.460; P < 0.015) and age (OR 1.065, 95% CI 1.016–1.116; P = 0.008) were independent determinants. In addition, the multivariate regression analysis performed to detect the presence of AC revealed that BAC (OR 1.448; 95% CI 1.011–1.136; P < 0.044) and age (OR 1.098; 95% CI 1.061–1.136; P < 0.001) were independent variables (Table 4).

**Table 4:** Independent predictors of coronary artery calcification and aortic calcification.

	CAC						AC					
	Univariate logistic regression			Multivariate logistic regression analysis			Univariate logistic regression			Multivariate logistic regression analysis		
Predict	Odds ratio	95% CI	P-value	Odds ratio	95% CI	P-value	Odds ratio	95% CI	P-value	Odds ratio	95% CI	P-value
Breast artery calcification	2.425	1.848–3.183	<0.001	1.647	1.103–2.460	0.015	4.332	2.756–6.812	<0.001	1.448	1.011–1.136	0.044
Diabetes mellitus	2.687	1.354–5.335	0.005				1.663	1.005–2.753	0.048			
Age, years	1.117	1.079–1.156	<0.001	1.065	1.016–1.116	0.008	1.121	1.094–1.149	<0.001	1.098	1.061–1.136	<0.001
Hypertension	1.221	0.645–2.311	0.540				1.268	0.849–1.896	0.246			
HDL-C, mg/dL	0.959	0.936–0.981	<0.001				0.978	0.964–0.992	0.002			
Hs-CRP, mg/dL	1.008	1.003–1.014	0.003				1.002	0.998–1.007	0.313			
Creatinine, mg/dL	2.506	1.268–4.952	0.008	1.834	1.009–3.334	0.047	1.191	0.802–1.767	0.386			

CAC: Coronary artery calcification; AC: aortic calcification, HDL: high-density lipoprotein, CRP: C-reactive protein.

## 4. Discussion

Mammography is a screening tool used for the early diagnosis of breast cancer, which has been proven to reduce mortality. Current guidelines recommend evaluation of women above 40 years of age by means of mammography once a year (1). BAC is a finding that is incidentally detected during mammographic assessment and is not associated with cancer risk. Studies have shown that BAC may be related to atherosclerosis, age, diabetes, and chronic renal disease (14–16).

CAC is associated with reduced vascular compliance, abnormal vasomotor response, and impaired myocardial perfusion (17,18). Alkaline phosphatase plays a central role in vascular calcification (14,15). Vascular smooth muscle cells produce matrix vesicles that control mineralization in the intima and media (16). In addition, cells such as the microvascular pericytes and the adventitial myofibroblasts have the potential to produce a mineralized matrix as well as osteoblastic differentiation (14). Inflammatory mediators and increased lipid accumulation in atherosclerotic lesions induce the osteogenic differentiation of the smooth vascular muscles.

Several studies have shown a strong correlation between CAC grade and plaque burden, severity of atherosclerosis, and future cardiac events (10,11,19). In addition, the presence of CAC is associated with poor prognosis, both in the general population and in patients who have undergone revascularization (20–21). Microcalcifications in the fibrous cap may lead to plaque rupture while calcific nodules may result in disruption of the fibrous cap and thrombosis (9,22). Obstructive fibrocalcific lesions secondary to recurrent plaque ruptures are associated with ischemic clinical outcomes. The presence of CAC also affects the results of therapeutic percutaneous intervention. An analysis that included 6296 patients showed that less complete revascularization was achieved and mortality was higher in patients with CAC (23).

The relation between cardiovascular events and BAC in the media layer has been particularly supported (24). Studies by Maas et al. (6) and Pecchi et al. (7) showed the correlation between CAC and BAC in Caucasian women. Newallo et al. (8) detected that BAC, when detected in African American women, was an independent variable for coronary artery calcium score (>100) and coronary artery stenosis (≥50%). Jiang et al. (25) published a metaanalysis that involved 10 cross-sectional studies and 3952 patients. This analysis showed that the presence of BAC was associated with a 3.86-fold increase in coronary artery disease (CAD) (95% CI 3.25–4.59). In our study, a 2.4-fold increase in CAC risk was detected for all grades of BAC. This correlation also maintained its determining characteristic in multivariate regression models.

The results from previous studies investigating the correlation between BAC and inflammation are controversial. In a study by Maas et al. (26), Hs-CRP and fibrinogen levels were similar in BAC-negative and BAC-positive patients. However, Pidal et al. (27) reported that BAC-positive patients had higher Hs-CRP levels. In our study, CRP measurement was performed with 286 patients, and the BAC-positive patients were observed to have markedly higher CRP levels. Furthermore, in our study, the CRP level showed a significant correlation with both BAC and CAC. 

AC reduces arterial elasticity and affects cardiovascular hemodynamics unfavorably (28). This is important when considering the wide range of conditions affected, which include arterial hypertension, aortic valve stenosis, limb ischemia, myocardial infarction, and congestive heart failure. Diffuse calcification causes an increased afterload that is secondary to reduced arterial compliance, resulting in left ventricular hypertrophy and reduced cardiac perfusion (29). Postmortem studies have revealed the association between aortic calcification and atherosclerosis (30). In a metaanalysis by Goncasves et al. (31), which included 11,250 patients, a notable increase in risk was observed for cardiovascular events, cerebrovascular events, and cardiovascular death. This is applicable to both medial and intimal atherosclerosis (32). In their study, Eisen et al. (33) found that cardiovascular events were markedly higher in stable ischemic heart disease patients with aortic calcification as compared to those without calcification. In our study, patients with BAC were observed to have notably higher AC rates and calcification severity. In addition, there was a good correlation between AC and CAC.

The most significant limitation of our study was the fact that clinical CAD data could not be analyzed. The interpretation of the results was made indirectly through CAC. This correlation may not have the same power as that of clinical events. In addition, our retrospective analysis included insufficient data on smoking, which is an important risk factor. Using a standard calcification scoring, such as Agatston, may have been more sensitive. However, simple visual grading, as previously described, was preferred because this method is simple, easy, and can be applied to a whole range of different CT protocols, including nongated CT. 

Our study revealed that both CAC and AC are more common in BAC-positive women. In addition, the grade of BAC was found to be an independent risk factor for CAC. These results suggest that positive BAC findings detected in women provide indirect insight into the presence of coronary atherosclerosis and plaque pathology.
